# Juvenile dermatomyositis (JDM) sine myositis

**DOI:** 10.1186/1546-0096-9-S1-P62

**Published:** 2011-09-14

**Authors:** C Schuetz, V Mohr, C Pfeiffer, A Schulz, KM Debatin

**Affiliations:** 1 Department of Pediatrics and Adolescent Medicine, Ulm University, Germany; 2 Department of Dermatology, Ulm University, Germany

## Background

A subgroup of JDM patients presents with an amyopathic form (sine myositis). Diagnosing this amyopathic form is challenging as classical JDM may begin with isolated cutaneous manifestations. Also there is no consensus on how to treat JDM sine myositis.

## Aim

Case presentation and discussion in the context of existing studies.

## Methods/case

6 y/o girl from Kazakhstan presenting with discrete heliotrope of upper eyelids, erythema of the chest and extensor surfaces of extremities, Gottron’s papules as well as scarred piecemeal necrosis on 1 finger and 2 toes. Close examination revealed nailfold tortuositas. Muscle strength was 5/5, CMAS 48/51. Inflammatory parameters were not elevated, transaminases, CK and aldolase in the normal range, ANA >1:2560. Follow-up over 12 months showed no progression or muscle involvement.

## Results

A recent MRI of the quadriceps femoris showed no signs of myositis. There was no improvement of skin changes following 6 months of continuous therapy with chloroquine, but no additional piecemeal necrosis.

## Conclusion

The data shows that the prevalence of patients with an amyopathic form of JDM varies between 4-20%. According to the literature, prognosis is excellent. However, there are no recognized predictive factors (e.g.biomarkers, imaging) to estimate progression to classical JDM with myositis: 25% of all patients with the initial diagnosis of JDM sine myositis develop muscle involvement over time. Also there is no consensus on optimal immunosuppressive therapy of these patients to avoid over- or undertreatment.

